# Methotrexate Gold Nanocarriers: Loading and Release Study: Its Activity in Colon and Lung Cancer Cells

**DOI:** 10.3390/molecules25246049

**Published:** 2020-12-21

**Authors:** Beatriz Álvarez-González, Marisa Rozalen, María Fernández-Perales, Miguel A. Álvarez, Manuel Sánchez-Polo

**Affiliations:** 1 Department of Inorganic Chemistry, Faculty of Science, University of Granada, Campus Fuentenueva s/n, 18071 Granada, Spain; abeatriz@correo.ugr.es (B.Á.-G.); mariafeper@correo.ugr.es (M.F.-P.); mansanch@ugr.es (M.S.-P.); 2 Department of Inorganic and Organic Chemistry, Faculty of Science, University of Jaén, Campus las Lagunillas s/n, 23071 Jaén, Spain; malvarez@ujaen.es

**Keywords:** Au nanoparticles, nanocarriers, methotrexate, anticancer drug, chemotherapeutics, controlled release

## Abstract

In the present study, the synthesis of gold nanoparticles (AuNPs) loaded with methotrexate (MTX) has been carried out in order to obtain controlled size and monodispersed nanocarriers of around 20 nm. The characterization study shows metallic AuNPs with MTX polydispersed on the surface. MTX is linked by the replacement of citrate by the MTX carboxyl group. The drug release profiles show faster MTX release when it is conjugated, which leads to the best control of plasma concentration. Moreover, the enhanced release observed at pH 5 could take advantage of the pH gradients that exist in tumor microenvironments to achieve high local drug concentrations. AuNP–MTX conjugates were tested by flow cytometry against lung (A-549) and colon (HTC-116) cancer cell lines. Results for A-549 showed a weaker dose–response effect than for colon cancer ones. This could be related to the presence of folate receptors in line HTC-116 in comparison to line A-549, supporting the specific uptake of folate-conjugated AuNP–MTX by folate receptor positive tumor cells. Conjugates exhibited considerably higher cytotoxic effects compared with the effects of equal doses of free MTX. Annexin V-PI tests sustained the cell death mechanism of apoptosis, which is normally disabled in cancer cells.

## 1. Introduction

Gold nanoparticles (AuNPs) have been investigated and established for its use as antineoplastic, anti-arthritic, antibacterial and antidiabetic agents. Moreover, AuNPs are an excellent delivery vehicle for different therapeutic agents, mainly anticancer drugs such as cisplatin, doxorubicin, tamoxifen, paclitaxel or methotrexate.

Their unique shape (spherical, rod shape, core shell, nanorods, etc.) with size varying from 1 to 100 nm, surface properties, inert nature, high biocompatibility and non-cytotoxicity represent important advantages. Furthermore, the large functional surface to mass ratio increases efficient drug payload, avoiding the cytotoxic effects in healthy tissues caused by overdoses [1]. The drug attachment can be accomplished by covalent binding, encapsulation, electrostatic absorption and other non-covalent assemblies [2].

MTX is an analogue of folic acid, where the main difference is that folic acid has a hydroxyl group at the 4-position of the pyridine ring while MTX presents an amine group at the same position. Due to the structural similarity, cells internalize MTX through similar transport systems as folates, inhibiting dihydrofolate reductase (DHFR), a critical enzyme in the folic acid cycle and key to regulating homeostasis, leading to reduced cell viability and cell death. As folate receptors are overexpressed on the cell membranes of many types of cancer cells, MTX has proven to be an effective targeting agent [3,4] and showed a potent anticancer effect. It has been used for the treatment of acute leukemia, osteogenic sarcoma, choriocarcinoma, breast cancer, pulmonary and epidermoid carcinoma and intratracheal chemotherapy. It is also used in bone marrow transplantation, severe psoriasis and rheumatoid arthritis [5]. However, its clinical application is limited due to its poor solubility, short half-life in the bloodstream and rapid diffusion throughout the body [6].

Existing studies [7,8,9,10,11,12] have shown that linking MTX to AuNPs allows for efficient and selective uptake, a property unique to nanocarriers, enhancing therapeutic efficacy and lowering effective doses. For instance, the effect of free MTX on lung cancer cells is seven times less than Au-MTX conjugated nanoparticles synthetized, as observed by Cheng et al. [8]. 

Controlled drug release can be achieved by: (i) passive targeting through accumulation based on the enhanced permeability and retention effect (EPR) observed for relatively large-sized nanoparticles (~15–100 nm) [13,14,15,16,17] in solid tumor models or (ii) active targeting using various payload release mechanisms including pH change [18,19] or triggering by endogenous glutathione utilizing thiol-to-thiol ligand exchange [20,21,22]. Because ligand exchange is based upon competitive affinity to the gold surface between the original and incoming ligands [21], MTX can be used not only as a drug but also as a potential targeting ligand [10] because it can be directly bound to AuNPs via carboxylic groups to form AuNP–MTX conjugates. In vitro cytotoxicity in lung tumor cells showed a higher accumulation of MTX conjugated with AuNPs compared with equal doses of free MTX. Moreover, AuNP-MTX conjugates suppressed tumor growth in a mouse ascites model of Lewis lung carcinoma.

Tran et al. [11] synthetized AuNPs using MTX as both reducing agent and capping molecule obtaining various sizes between 3 and 20 nm. To examine anticancer effects, they applied lactate dehydrogenase (LDH) and MTT assays, demonstrating that small-sized (3 nm) AuNP-MTX conjugates exhibited considerably high cytotoxic effects on human choriocarcinoma cell lines compared to the effects of equal doses of free MTX

Murawala et al. [10] investigated the efficiency of MTX-loaded BSA-capped AuNPs (Au-BSA-MTX) in preventing the propagation of breast cancer cell lines (MCF-7) based on MTT and Ki-67 proliferation assays. 

Bessar et al. [7] designed water-soluble gold nanoparticles functionalized by sodium 3-mercapto-1-propansulfonate (Au-3MPS) and loaded with MTX, in order to improve the solubility, stability and biodistribution of the drug for psoriasis treatment. They showed a fast release (80% in an hour) and were tested successfully on normal C57BL/6 mouse skin to trace the absorption behavior.

Wang et al. [12] synthetized Au-MTX conjugates using a hydrothermal growth method to lead into nanochains and discrete nanoparticles of around 30 nm. Compared with nanochains, the discrete nanoparticles showed almost equal drug loading capacity (between 8.6% and 25.1%) but higher control of drug release, colloidal stability and in vitro anticancer activity (A-549 cell line/lung cancer).

Despite the progress which has been achieved, AuNP synthesis still presents some drawbacks, such as monodispersity, unsuccessful functionalization and stability, lack of size homogeneity, limited opsonization or plasma and blood internalization. 

Against this background, the main objective of the present study is to synthesize and characterize discrete, controlled-size methotrexate gold nanoparticles (AuNP–MTX), using citrate as a reduction agent, to be used as nanocarriers of MTX. On one hand, the anticancer activity has been tested by studying the in vitro cytotoxicity of lung (A-549) and colon cancer (HTC-116) cell lines. On the other hand, we have proposed an AuNP–MTX formation mechanism and fitted the drug release dataset to a pharmacokinetic model to show how MTX concentration is maintained in blood or in target tissues.

## 2. Results and Discussion

### 2.1. Characterization of AuNPs and AuNP–MTX Conjugates

#### 2.1.1. UV-Vis Spectroscopy

**Figure 1:**
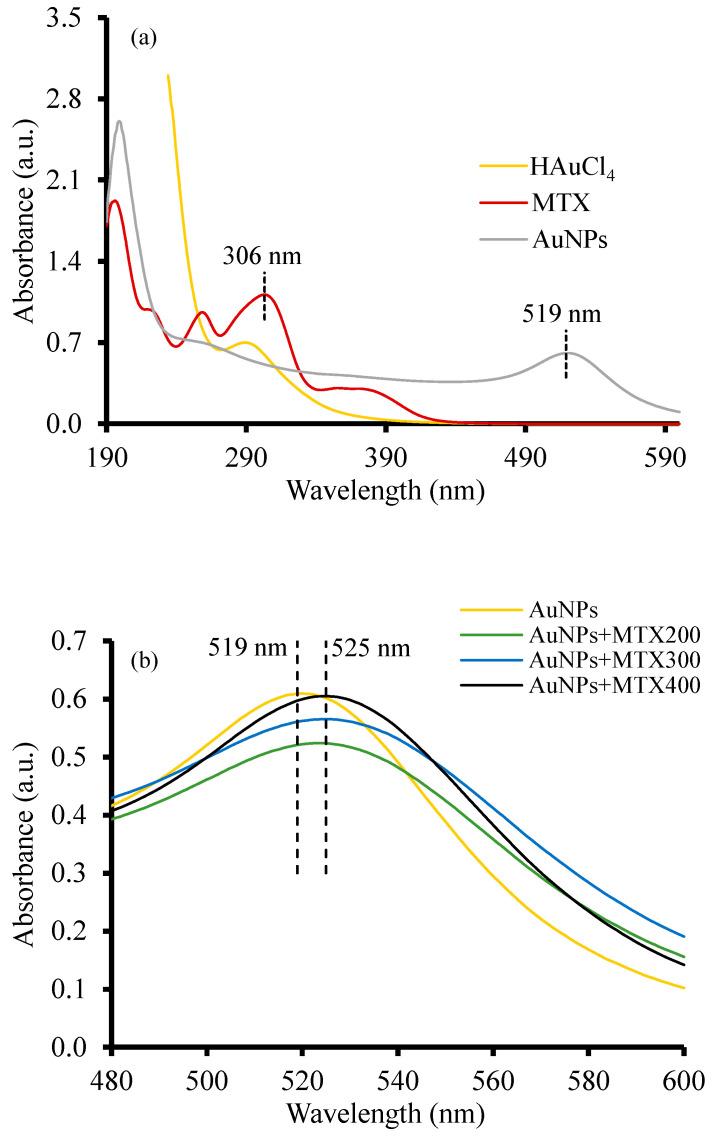
(**a**) Comparative UV-Vis absorption spectra of free MTX, HAuCl_4_ and AuNPs. (**b**) Absorption spectra of AuNP–MTX conjugates with different concentrations of MTX.

The optical properties of the as-prepared AuNPs and AuNP–MTX conjugates measured by UV-Vis adsorption are depicted in Figure 1. Free MTX exhibits three characteristics.

Absorption peaks were centered at 258, 306 and 372 nm (Figure 1a). HAuCl_4_ presented a peak around 300 nm which disappeared, with a new peak emerging at 519 nm corresponding with the surface resonance plasmon band (SPR) of AuNPs [9,10,12,23]. Figure 1b shows a bathochromic shift around 525 nm for the AuNP–MTX conjugates, which indicates an increase in size particle [24] and also a modification in the nanoparticle’s surface [25]. This could be due to the chemical absorption of MTX into the AuNP’s surface, justifying the band shift from 519 to 525 nm [8,9,10,12].

#### 2.1.2. X-ray Diffraction and FTIR Analysis

**Figure 2:**
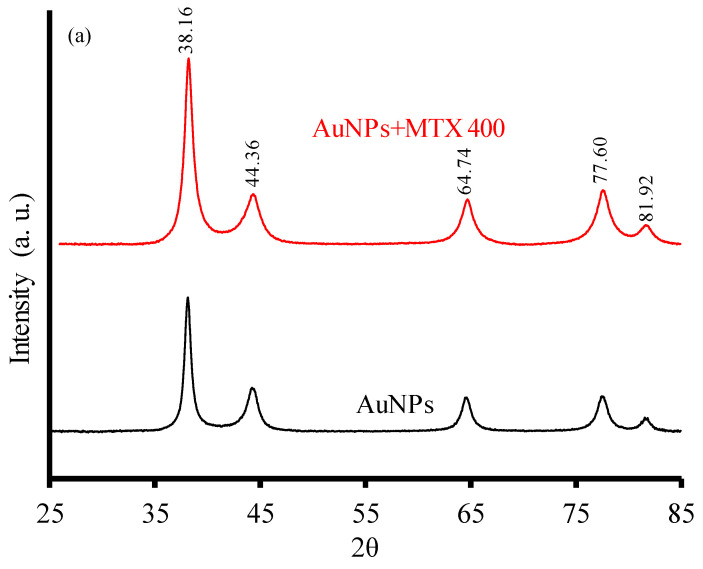

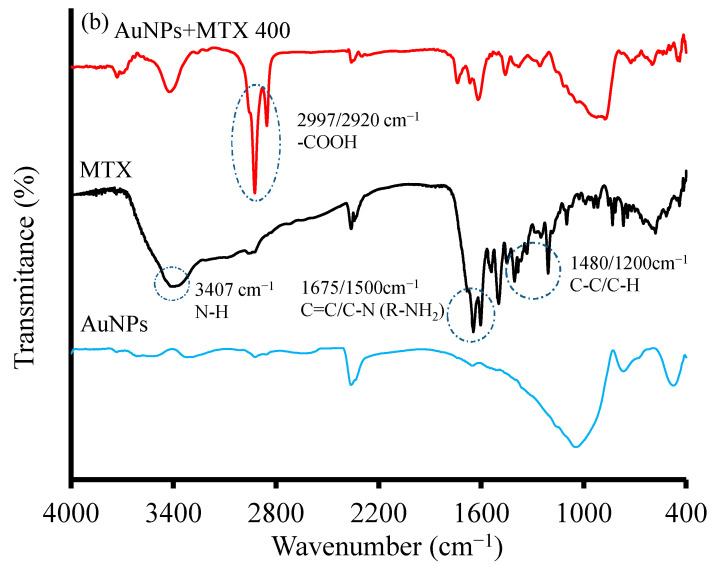
(**a**) XRD diffractograms of AuNPs and AuNP–MTX400. (**b**) FTIR spectra of free MTX, gold nanoparticles (AuNPs) and MTX conjugates (AuNP–MTX400).

AuNPs and AuNP–MTX conjugates revealed the same typical XRD pattern (Figure 2a) characterized by five diffraction peaks located at 38.16° (1 1 1), 44.36° (2 0 0), 64.74° (3 1 1), 77.6° (3 1 1) and 81.92° (2 2 2), confirming a gold crystalline phase with face centered cubic (FFC) geometry (JCPDS 04-0784).

The FTIR spectra of free MTX, AuNPs and AuNP–MTX400 are shown in Figure 2b, confirming the formation of MTX conjugate. The peak at 3407 cm^−1^ for free MTX indicates the presence of an NH group. Absorptions at 2997 and 2921 cm^−1^ correspond with the existence of a carboxylic group. Peaks in the region of 1675–1500 cm^−1^ can be attributed to C=C vibration in the aromatic ring and R-NH_2_ vibrations, while peaks at 1480 and 1200 cm^−1^ represent the stretching vibration of the C-C or C-H bonds [26]. Compared with free MTX, the FTIR spectrum of AuNP–MTX conjugates exhibits similar peaks as a consequence of the interaction between AuNPs and MTX, indicating the successful uploading of MTX into the gold nanoparticles, as observed previously in the literature [7,11].

#### 2.1.3. HRTEM Microscopy

**Figure 3:**
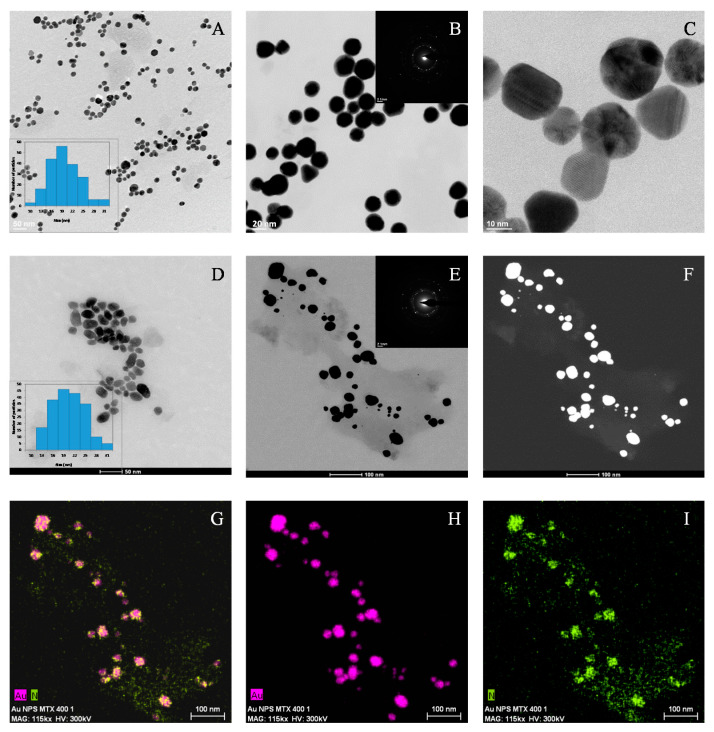
(**A**) HAADF image of a representative group of AuNPs. (**B**) SAED image showing the presence of a gold nanocrystalline phase. (**C**) HRTEM lattice fringe image of AuNPs showing multiple domains. (**D**) HAADF image of a representative group of AuNP–MTX conjugates. (**E**) Dark field image of AuNPs-MTX conjugates. SAED image showing the presence of a gold nanocrystalline phase. (**F**) Maps for relative distribution of the elements (pink = Au and green = N) jointly shown (**G**–**I**).

The morphology of synthesized AuNPs and AuNP–MTX conjugates was examined by HRTEM analysis. Images in Figure 3A–C confirm the formation of homogeneous-sized, mostly spherical and highly dispersed gold nanoparticles. 

The calculated average size was 18.40 ± 4.75, with a narrow distribution range between 7 and 19 nm, as shown in the histogram (Figure 3A). The SAED image (Figure 3B) confirmed the presence of a crystalline gold phase, with distances of 2.35 and 2.09 Å corresponding with the (1 1 1) and (2 0 0) planes, which is in accordance with the XRD results. Finally, high-resolution images (Figure 3C) showed polycrystalline domains for spherical particles [27].

**Figure 4:**
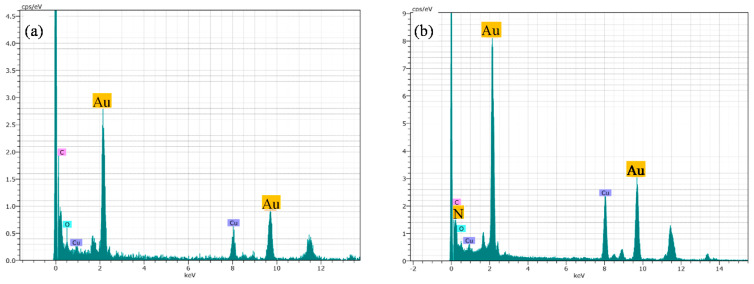
(**a**) EDX analysis of AuNPs confirmed the presence of gold. (**b**) EDX analysis for AuNP–MTX400 confirmed the presence of N as indicator of MTX presence.

The addition of MTX does not lead to significant changes regarding morphology or size, as only a slight SPR shift from 520 to 525 nm is observed. The representative sample of AuNP–MTX400 confirms the formation of spherical, monodispersed nanoparticles with a similar average size of 19.32 ± 4.96 nm and distribution range between 10 and 31 nm (Figure 3D). The crystalline nature is also observed in the SAED image (Figure 3E), where a group of gold nanoparticles appeared covered with MTX. This is confirmed with the chemical element mapping analysis (Figure 3G–I), highlighting the homogeneous distribution of Au and N (as an indicator of the presence of MTX) in the sample. N was not distributed in layers but along the sample, which suggested the formation of AuNP–MTX conjugates. EDX analysis (Figure 4b) also confirmed the presence of gold and nitrogen, as an indicator of the presence of MTX. 

#### 2.1.4. X-ray Photoelectron Spectroscopy (XPS)

**Figure 5:**
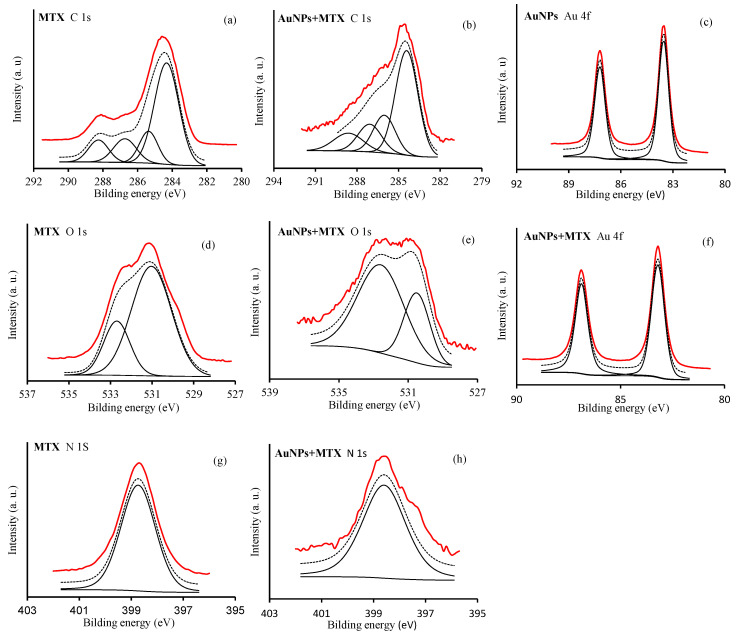
XPS spectra of regions C 1s: (**a**,**b**); O 1s: (**d**,**e**); N 1s: (**g**,**h**) and Au 4f: (**c**,**f**) of MTX, AuNPs and AuNP–MTX400.

Chemisorption of MTX on the surface of AuNPs was explored through the XPS spectra of MTX in pure solid form as well as in the adsorbed condition (AuNP–MTX400). Figure 5 shows the high-resolution XP spectra in the C 1s, O 1s, N 1s and Au 4f regions for free MTX, AuNPs and AuNP–MTX400. 

For free MTX, in the C 1s region (Figure 5a), four peaks were identified at: (i) 284 eV, corresponding with sp^2^ hybridized carbon (C=C); (ii) 285.4 eV, corresponding with sp^3^ hybridized carbon (C-C) and C-N bonds; (iii) 288.4 eV, assigned to C-O bonds from hydroxyl/ether groups; (iv) 288.4 eV, assigned to carboxyl (-COOH) moiety. Concerning the O 1s region (Figure 5d), two peaks are identified around 530.5 and 532 eV corresponding to the oxygen atoms in the carbonyl ring (>C=O) and the carboxyl (-COOH) moieties, respectively [28,29], whereas in the N 1s region, only a peak at 398.7 eV was identified (Figure 5g). Otherwise, XPS spectra in the Au 4f regions showed two peaks with a bond energy of 83.5 and 87.2 eV, assigned to Au 4f_7/2_ and Au 4f_5/2_, respectively (Figure 5c).

After the adsorption of MTX on the AuNP’s surface, the C 1s peak located at 288.2 eV and derived from the carboxylic carbon is shifted until 288.7 eV and can be attributed to the carboxylate group, -COO- (Figure 5b). This is in accordance with the results obtained by [8], providing evidence that MTX is adsorbed chemically on the AuNP’s surface in carboxylate form. Moreover, the oxygen peak in the carboxylate group results in the shift to lower bonging energies, as a consequence of the increase in electron density of oxygen on the carboxylate group with respect to the carboxylic group (Figure 5e). The Au 4f and N 1s regions remained unchanged (Figure 5f,h). Based on the XPS results and the hypothesis of Chen et al. [8], MTX exchanges with the citrate molecules on the AuNP’s surface and bonds to the AuNP’s surface through a covalent bond.

Covalent bonding of therapeutic agents on nanocarriers is usually favored because the bond strength makes the NP drug conjugates highly stable and therefore is most likely to be disrupted only under harsh environments inside lysosomes [30]. MTX-conjugated NPs were believed to be taken up into cells to a greater extent by the human folate receptor than free MTX [8].

### 2.2. Stability Analysis of AuNP–MTX Conjugates

**Figure 6:**
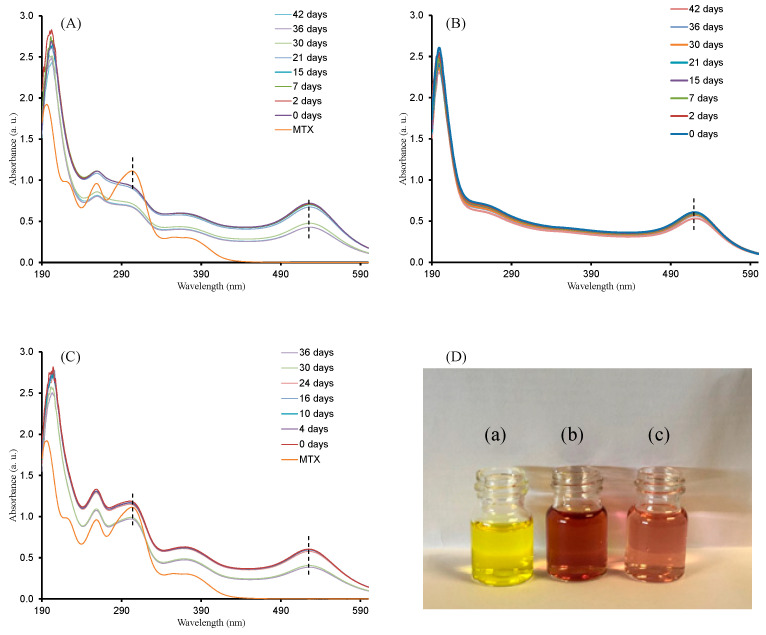
Colloidal stability test for: (**A**) AuNP–MTX400; (**B**) AuNP–MTX200 and (**C**) AuNPs. (**D**) Short-term stability (24 h) in PBS with 10% FBS of HAuCl4 (**a**), AuNP–MTX400 (**b**) and AuNPs (**c**) included as a reference. In all cases, they are not stable when diluted by only phosphate-buffered solution (PBS), while in the presence of fetal bovine serum (FBS), the particles are stable at least for 24 h.

Synthesized gold colloids with different amounts of MTX where tested for long-term colloidal stability by using UV-Vis absorption spectroscopy. Samples were stored in darkness at 4 °C. As observed in Figure 6, AuNPs and the three AuNP–MTX conjugates maintained the position of surface plasmon resonance (519 and 525 nm, respectively) and intensity for more than one month. MTX peaks around 280 and 308 nm also remained unaltered, confirming the stability of the synthetized conjugates. This is in accordance with the results obtained in other studies [24].

The short-term study confirmed that the conjugates were not stable when diluted by only phosphate-buffered solution (PBS), while in the presence of fetal bovine serum (FBS), the particles were stable at least for 24 h without the addition of DMEM. As might be expected, this implies that AuNPs and synthetized AuNP–MTX conjugates are sensitive to high salt content, but FBS can stabilize the nanoparticles and maintain their dispersity. 

### 2.3. Quantification and Drug Release Study

The amount of MTX conjugated on the AuNP’s surface is 15.1%, 19.4% and 23.6% for the samples AuNP–MTX200, AuNP–MTX300 and AuNP–MTX400, respectively. These amounts are slightly higher than those obtained by Wang et al. [12].

**Figure 7:**
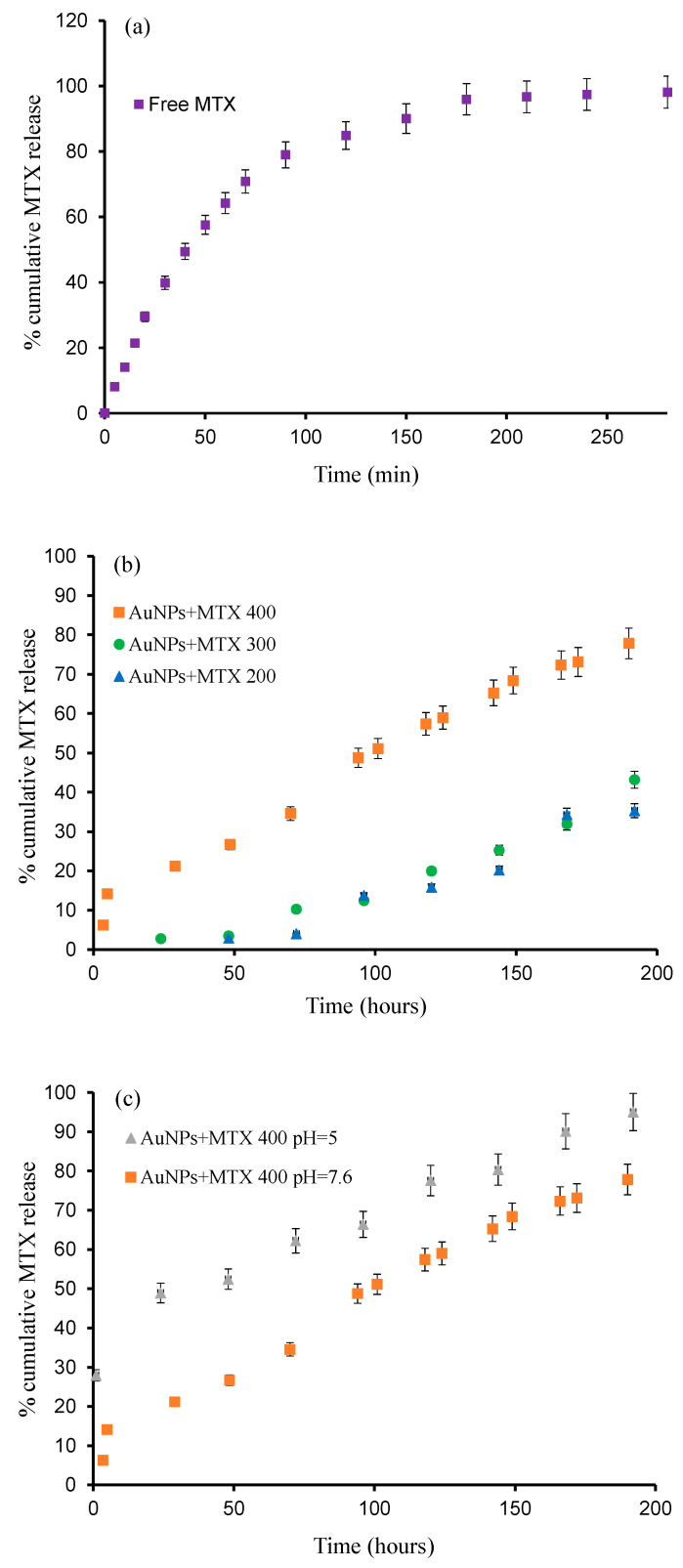
The release profiles of: (**a**) Free MTX; (**b**) AuNP–MTX 400, AuNP–MTX 300 and AuNP–MTX 200; (**c**) AuNP–MTX 400 at pH 7.6 and pH 5.

In vitro release profiles of MTX from AuNPs prepared at different concentrations have been investigated and are shown in Figure 7. As observed, free MTX is released until 80% in the first 4 h, reaching 100% by 8 h (Figure 7a), but when MTX is conjugated with AuNPs, the release time increases until 200 h (Figure 7b). The obtained percentage of released drug increases following the sequence: AuNP–MTX400 (78%) > AuNP–MTX300 (43%) > AuNP–MTX200 (35%).

The results presented in Figure 7c show a clear pH effect, with a faster release at pH 5 (95% after 200 h) compared with the 77% reached at pH 7.6. Consequently, at the tissue level, synthetized AuNP–MTX400 could take advantage of the pH gradients that exist in tumor microenvironments to achieve high local drug concentrations, as has been observed in other studies [31,32]. Finally, at the intracellular level, pH-responsive AuNPs also could escape from the acidic endo-lysosomal compartments for cytoplasmic drug release [33,34], where MTX could inhibit the DHRF enzyme, breaking the folic acid cycle [30].

### 2.4. Kinetic Modeling

The mechanisms of MTX conjugation have been tested by applying different kinetic models, in order to fit the experimental cumulative drug release data (Table 1). 

The release of free MTX fitted a first-order model represented by the following equation:

(1) $$\mathrm{logM}={\mathrm{logM}}_{0}-k\cdot \frac{t}{2.303}\;$$

where Mo is the initial concentration of drug, k is the first-order rate constant and t is the experiment time. 

MTX released from AuNP–MTX200, AuNP–MTX 300 and AuNP–MTX400 followed a zero-order model, representative of drug dissolution from dosage forms that do not disaggregate and release the drug slowly, following the equation:

(2) $$Q_{t}=Q_{0}+{\;K}_{0}\cdot t\;$$

where Q_t_ is the amount of drug dissolved in time t, Q_0_ is the initial amount of drug in the solution and K_0_ is the zero-order release constant expressed in units of concentration/time.

Finally, the MTX released at acid fitted the Hixson–Crowell model, represented by the equation:

(3) $$W_{t}^{1/3}=W_{0}^{1/3}-\;\kappa \cdot t\;$$

where *W*_0_ is the initial amount of drug in the pharmaceutical dosage form, *W_t_* is the remaining amount of drug in the pharmaceutical dosage form at time *t* and κ is a constant incorporating the surface–volume ratio.

The dataset for free MTX was fitted to a first-order kinetic model, obtaining a value of k = 0.0026 min^−1^ with a regression coefficient of r^2^ = 0.988, while AuNP–MTX200, AuNP–MTX300 and AuNP–MTX400 were fitted to a zero-order kinetic model, obtaining values of k = 0.0034, 0.004 and 0.006 mg/min and regression coefficients of r^2^ = 0.9116, 0.9757 and 0.9801, respectively. Obtaining a zero-order release, in which a drug is released at a constant rate, is the ultimate goal of all controlled-release drug delivery mechanisms. It leads, in principle, to the best control of plasma concentration and offers several advantages, including improved patient compliance and reduction in the frequency of drug administration [35]. Finally, the acid pH release of AuNP–MTX400 was fitted to the Hixson–Crowell model, obtaining a value of κ = 0.0002 mg^1/3^/min with a regression coefficient of r^2^ = 0.9559.

According to the results obtained, we selected the sample AuNP–MTX400 for the in vitro anticancer activity tests.

### 2.5. In Vitro Anticancer Activity

The cytotoxic effect of the free MTX, AuNPs and AuNP–MTX400 were tested against colorectal cancer (HTC-116) and human lung carcinoma (A-549) cell lines. The dose–response curve for free MTX showed IC_50_ values of 2.3, 0.37 and 0.15 mM after 12, 24 and 48 h for the HTC-116 cell line (Table 2).

The results obtained for lung cancer (A-549) do not show any effect at the concentrations studied after 12 or 24 h; however, after 48 h, the IC_50_ calculated is 0.10 mM, reflecting a lower sensitivity and slower response time for this cell line.

**Figure 8:**
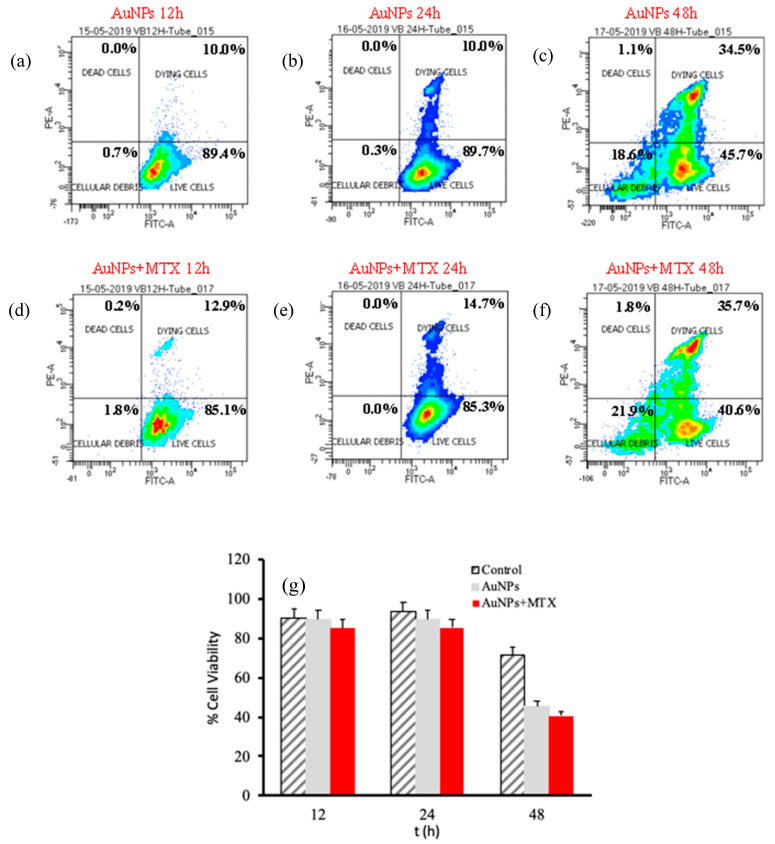
Percentage of cell viability for AuNPs and AuNPs-MTX400 equivalent cocnetrations obtained by flow cytometry for the A-549 cell line after 12 (**a**–**d**), 24 (**b**–**e**) and 48 (**c**–**f**) incubation hours. (**g**) Calculated % cell viability for equivalent concentrations of AuNPs and AuNPs-MTX400.

Interestingly, the results obtained for the A-549 cell line showed a weaker dose–response effect (Figure 8). This could be related to the presence of folate receptors in line HTC-116 in comparison to line A-549, supporting the specific uptake of folate-conjugated AuNP–MTX by folate receptor positive tumor cells. This statement is in agreement with a recent study by Soe et al. [36]. Their in vitro study revealed enhanced cellular uptake and apoptosis in folate receptor-expressing colorectal cancer cells (HCT-116 and HT-29) as compared to that in lung cancer cells (A549), which do not express folate receptors.

In accordance with this hypothesis, results obtained for the A-549 cell line for AuNPs as well as AuNP–MTX400 did not show a significant effect by decreasing the percentage of live cells at 12 or 24 hours of exposure (Figure 8). After 48 h, AuNPs and AuNP–MTX400 showed significant cytotoxic activity, decreasing the percentage of live cells to 28.5% and 25.9% respectively. As recently demonstrated and tested by estimating the protein expression of apoptotic signaling proteins, AuNPs by themselves acts as an anticancer agent against lung carcinoma cell line A-549 [37]. 

**Figure 9:**
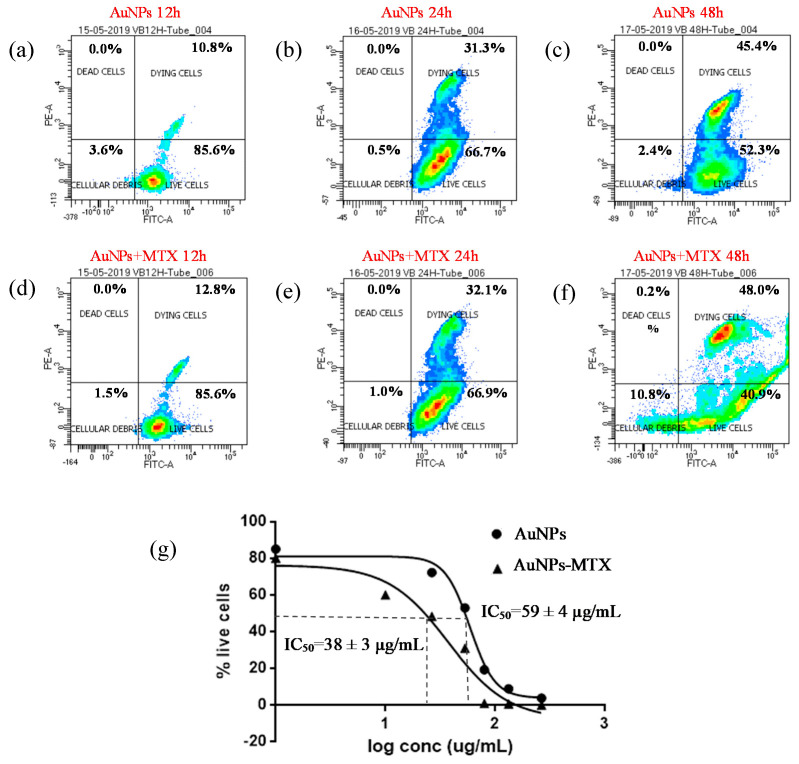
Percentage of cell viability for AuNPs and AuNP–MTX400 equivalent concentrations obtained by flow cytometry for the HTC-116 cell line after 12 (**a**–**d**), 24 (**b**–**e**) and 48 (**c**–**f**) h of incubation. (**g**) Calculated IC_50_ for equivalent concentrations of AuNPs and AuNP–MTX400 after 48 h of incubation.

For the HTC-116 cell line, AuNPs showed a significant effect, decreasing the % live cells from 85.6% to 52.3% after 12 and 48 hours of exposure, respectively (Figure 9a,c). For AuNP–MTX400, this percentage decreased to 40.9% after 48 hours of exposure. This is confirmed by the IC_50_ calculated (Figure 9g), which also decreased from 70 µg/mL for AuNPs to 37.5 µg/mL when MTX was conjugated with AuNPs, lowering the IC_50_ to half of the free MTX and confirming the therapeutic effect of the conjugate for this cell line. 

**Figure 10:**
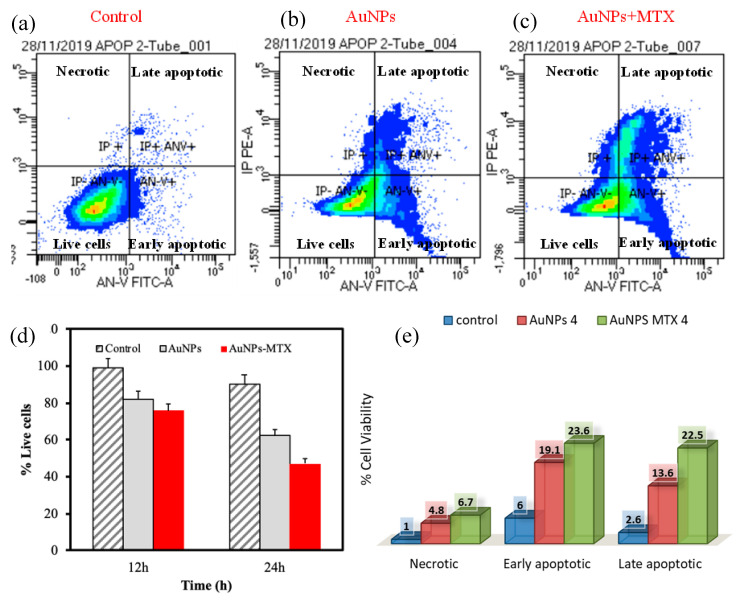
Flow cytometric analysis to determine death modes of cancer cells to HTC-116. The percentage of necrotic and apoptotic HTC-116 cells (**a**) Control (**b**) AuNPs and (**c**) AuNP–MTX400. (**d**) Comparison of time response to equal doses of AuNPs and AuNP–MTX400. (**e**) Representative percentage of necrotic and apoptotic AuNPs and AuNP–MTX400.

Finally, to determine the level of apoptosis induced by AuNPs and AuNP–MTX400, flow cytometry density plots showing annexin V (X-axis) and propidium iodide (Y-axis) staining of HTC-116 cells (Figure 10) were constructed. The right lower quadrant represents annexin V positive/propidium iodide (PI) negative staining, indicating early apoptosis. The right upper quadrant represents both high annexin V and PI staining, indicating late apoptosis, and the left upper quadrant represents low annexin V and high PI staining, indicating necrosis. The left lower quadrant indicates viable cells. The representative density plots demonstrate dual staining after no treatment (Figure 10a), treatment with AuNPs (Figure 10b) and the same concentration treatment with AuNP–MTX400 (Figure 10c). This latter exhibited improved cytotoxicity compared to the individual AuNPs at all tested concentrations. Finally, the data presented in Figure 10e indicate that cell death occurs mostly from apoptosis to necrosis for the cell line HTC-116. As most tumor cells have presumably disabled apoptosis to achieve a malignant state, cancer cells should be more resistant to DNA-damaging anticancer agents than the normal cells from which they arise [38]. In conclusion, the AuNP–MTX conjugates tested in this study showed an improved cytotoxic effect against the HTC-116 cell line compared with free MTX, inducing apoptosis as the main mechanism of cell destruction, which could be a major contributor to anticancer therapy-induced killing of tumor cells.

## 3. Materials and Methods 

### 3.1. Reagents

Chloroauric acid (HAuCl_4_), trisodium citrate dehydrate (Na_3_C_6_H_5_O_7_), methotrexate (C_20_H_22_N_8_O_5_) and sodium hydrogen carbonate (NaHCO_3_) were of analytical grade and used without further purification (Sigma-Aldrich, St. Louis, MO, USA). All solutions were made using double distilled Milli-Q water (18 MΩ).

### 3.2. Synthesis of Gold Nanoparticles (AuNPs)

Reference gold nanoparticles were synthesized based on the methods described in detail elsewhere [12,39]. Briefly, 1 mL of chloroauric acid dissolution (25 mM) was added to 150 mL of a boiling sodium citrate solution (TCS) (2.2 mM) under vigorous stirring and kept in the dark for 12 min at 90 °C. The mixture color quickly changed from pale yellow to light red, indicating the generation of AuNPs. Finally, the resulting colloid was cooled at room temperature, centrifuged at 12,000 rpm for 15 min and dried at 60 °C for 12 h.

### 3.3. Synthesis of Gold Nanoparticles with Various MTX Contents (AuNP–MTX)

One-step synthesis was undertaken by adding different amounts of a 10 mM MTX solution, made in 1 mM K_2_CO_3_, to the AuNP solution prepared previously and kept for another 10 min at 90 °C. Finally, the resulting colloid was cooled at room temperature, centrifuged at 12,000 rpm for 15 min and dried at 60 °C for 12 h. Different concentrations of AuHCl_4_ and MTX were tested with the aim of determining the optimal ratio (Table 3).

### 3.4. Characterization of AuNPs and AuNP–MTX Conjugates

The reduction of HAuCl_4_ and the formation of AuNPs were monitored by observing the changes in absorption spectra centered at 520 nm originating from the surface plasmon resonance of the AuNPs using an UV-visible spectroscopy with a VWR UV-1600PC UV/VIS spectrophotometer (VWR international GmbH, Darmstadt, Germany). Samples were analyzed in the 400–900 nm spectral range. The crystalline structure of AuNPs was examined using a BRUKER D8 ADVANCE diffractometer (Kα Cu) with a LINEXE detector (Bruker, Rivas-Vaciamadrid, Madrid, Spain). The chemical structure and functional groups of the AuNP–MTX were analyzed by Fourier transform infrared spectroscopy (FT-IR, NICOLET 20SXB spectrometer, Nicolet, Madison, WI, USA) and recorded in absorbance mode in the 4000–400 cm^−1^ range using a spectral resolution of 0.5 cm^−1^.

X-ray photoelectron spectroscopy (XPS) experiments were conducted on a Kratos Axis Ultra-DLD spectrometer equipped with Al Kα source (Kratos Analytical Ltd., Kyoto, Japan). CasaXPS software (version 2.3.16, Casa Software Ltd., Teignmouth, UK) was used to evaluate XPS data. 

The size and morphology of AuNPs and AuNP–MTX were studied with high-resolution transmission electron microscopy (HRTEM, FEI Company, Hillsboro, OR, USA) images, obtained using an FEI Titan, operated at 300 kV. SAED patterns were collected using a 10 µm aperture allowing collection of diffraction data from a circular area. Compositional maps of selected areas were acquired in scanning transmission electron microscopy (STEM, FEI Company, Hillsboro, OR, USA) mode using a Super X EDX detector (FEI, Hillsboro, OR, USA), formed by four windowless SSD detectors. STEM images were collected with a high angle annular dark field HAADF (FEI Company, Hillsboro, OR, USA) detector.

### 3.5. Short- and Long-Term Stability Test

First, 1 mL of gold colloid solution was mixed with 2 mL of milli Q water, PBS buffer (pH 7.4), Milli-Q water containing 10% FBS and PBS containing 10% FBS. After incubation at 37 °C for 1, 6, 12 and 24 h, the changes in the maximum absorption wavelength were determined by UV-vis spectroscopy (VWR international GmbH, Darmstadt, Germany). 

The long-term storage stability (one month) of the prepared silver nanoparticles conjugated with different amounts of methotrexate was also assessed after synthesis, keeping samples in darkness, refrigerated at 4 °C.

### 3.6. Drug Loading Capacity

The efficiency of drug conjugation was calculated by a direct method using the absorption of the MTX at 306 nm. Briefly, the unknown drug concentration of the nano-carrier system was determined using a calibration curve based on a series of known MTX concentrations. The drug conjugation efficiency was then calculated using the following equation [12]:

(4) $$\mathrm{Drug}\;\mathrm{loading}\;\%\;=\frac{\mathrm{Initial}\;\mathrm{free}\;\mathrm{MTX}\;\mathrm{added}\;\left(g\right)-\mathrm{MTX}\;\mathrm{measured}\;\mathrm{supernant}\;\left(g\right)}{\mathrm{weight}\;\mathrm{of}\;\mathrm{AgNPs-MTX}\;\left(g\right)}$$

### 3.7. pH-Dependent Drug Release Study

The release profiles for gold nanoparticles conjugated with different amounts of MTX (AuNPs–MTX 200, 300 and 400) were determined using dialysis bags (MWCO 8–14 kDa) with phosphate-buffered saline (PBS) as release media. First, 3 mg of AuNPs–MTX conjugates was weighed and introduced into the dialysis bags within 3 mL of PBS and immersed in 80 mL of PBS. The system was shaken at a speed of 150 rpm and incubated at 37 °C. At desired time intervals, 3 mL of solution was withdrawn and replaced with an equal volume of fresh buffer. MTX release was then quantified by measuring its absorbance using absorption spectroscopy at 306 nm and the concentration of the drug estimated with the aid of a standard curve. As a control experiment, the release of free MTX from the dialysis bag was also measured. 

Finally, the pH effect (pH 7.6 and 5) was studied for the highest concentration conjugated sample (AuNPs–MTX400). The selected conditions simulate physiologic environment (pH 7.4 and 37 °C) and endosomal/lysosomal compartment and cancer tissue environment in vitro (pH 5, 37 °C) [40,41].

### 3.8. Cytotoxicity and Anticancer Effect

In vitro bioassays were undertaken with colorectal cancer (HTC-116) (ECACC N°: 91,091,005 (lot N° 05K025) and human lung carcinoma (A-549) (ATCC N°: CCL-185 (lot N° 3624224) cell lines, obtained from the CIC cell bank of the University of Granada. Cell viability was determined through flow cytometry using a simultaneous double-staining procedure with fluorescein diacetate (FDA) and propidium iodide (PI) in the presence of free MTX, AuNPs and AuNPs–MTX conjugates. In total, 40,000 cells were separately incubated and distributed in 12-well plates for further 24 h incubation at 37 °C in a humid atmosphere enriched with 5% CO_2_. Three replicates of each experiment were performed to provide statistically significant support. The medium was removed, and fresh medium was added together with free MTX (at concentrations of 45.4, 454, 1136 and 4544 µg/mL), AuNPs (887, 1330 and 2260 µg/mL) and AuNPs–MTX (1420, 2130 and 4260 µg/mL). After 12, 24, and 48 h of treatment, 100 μL/well of propidium iodide solution (100 μg/mL) was added and incubated for 10 min at 28 °C in darkness. Afterwards, 100 μL/well of fluorescein diacetate (100 ng/mL) was added and incubated under the same aforementioned conditions. Finally, cells were recovered by centrifugation at 1500 rpm for 10 min and the precipitate was washed with PBS. Flow cytometric analyses were performed with a FACS Vantage™ flow cytometer (Becton Dickinson, Le Pont-de-Claix, France). The percentage viability was calculated in comparison to the control culture. The IC_50_ values were calculated using linear regression analysis from the Kc values at the employed concentrations using the software GraphPad Prism 6 (GraphPad Software, San Diego, CA, USA).

The extent of apoptosis was measured through an annexin V-fluorescein isothiocyanate (FITC) apoptosis detection kit as described in the manufacturer’s instructions (eBioscience, San Diego, CA, USA). The in vitro bioassay was undertaken with colorectal cancer (HTC-116). Then, 10,000 cells were separately incubated and distributed in 12-well plates for further 24 h incubation at 37 °C in a humid atmosphere enriched with 5% CO_2_. The medium was removed, and fresh medium was added together with AuNPs and AuNPs–MTX solutions at different concentrations. After 12, 24 and 48 h of treatment, 5 μL/well of annexin V was added and incubated for 15 min in darkness. Afterwards, 5 μL/well of propidium iodide was added and incubated under the same aforementioned conditions. Finally, cells were recovered by centrifugation at 1500 rpm for 10 min and the precipitate was washed with PBS. Flow cytometric analyses were performed with a FACS Vantage™ flow cytometer (Le Pont-de-Claix, France).

### 3.9. Statistical Analysis

Statistical analysis was performed by using the Graph Pad Prim v.6 software (GraphPad Software, San Diego, CA, USA). The one-way analysis of variance (ANOVA) statistical method was used to evaluate the significance of the experimental data. A value of *p* < 0.05 was considered statistically significant.

## 4. Conclusions

Controlled-sized, polydispersed AuNPs–MTX conjugates, with an average size of 19.32 ± 4.96 nm and distribution range between 10 and 31 nm, have been successfully synthesized, controlling temperature and pH and using citrate as a reducing agent. 

HRTEM mapping showed a homogeneous distribution of Au and N (as an indicator of the presence of MTX) with prevalence of a gold nanocrystalline phase (determined by SAED images), which suggests the formation of an AuNPs–MTX conjugate. XPS results confirmed as the formation mechanism the chemisorption of MTX through a carboxylic group (-COOH) onto AuNPs via exchange with a citrate molecule.

The calculated drug loading capacity for AuNPs–MTX400 reached 23.6%, releasing 78% of the initial MTX loaded onto the nanoparticles. MTX conjugated onto AuNPs not only decreased the drug release rate from around 4 h to 200 h but also changed from first-order to zero-order kinetic model, allowing a better control of plasma concentration and offering several advantages, including a reduction in the frequency of drug administration. The drug release rate also showed a pH stimuli response at acidic pH level (5) which could improve penetration into the tumor mass.

The anticancer activity tested in colon and lung cancer cells suggests the effectiveness of drug-conjugated AuNPs, enhancing the therapeutic effect by inducing apoptosis and decreasing the effective doses by half of MTX in the colon cancer cell line, with folate receptor, confirming the MTX mechanism of internalization, linking to dihydrofolate reductase (DHFR) and interrupting the folic acid cycle, which is usually overexpressed in cancer cells.

## Figures and Tables

**Table 1:** Calculated parameters and correlation coefficients (r^2^) of different models of release kinetics of AuNPs and the corresponding MTX conjugates synthesized in this study. Bold numbers point out best fitting values

Model	First Order		Zero Order		Hixson–Crowell	
	r^2^	k_H_ (h^−1^)	r^2^	k_H_ (h)	r^2^	k_H_ (h)
Free MTX	**0.9885**	0.9111	0.9609	0.9009	0.946	0.013
AuNP–MTX200	0.8857	0.0025	**0.9116**	0.0034	0.8952	0.00006
AuNP–MTX300	0.9409	0.0035	**0.9757**	0.004	0.9553	0.00008
AuNP–MTX400	0.8428	0.0112	**0.9801**	0.006	0.9594	0.0002
AuNP–MTX400 pH	0.933	0.0128	0.8632	0.0066	**0.9559**	0.0002

**Table 2:** IC_50_ values of free MTX at 12, 24 and 48 h for cell lines HTC-116 and A-549.

(MTX) IC_50_		HTC-116		(MTX) IC_50_		A-549	
Time	mM		µg/mL	Time	mM		µg/mL
12 h	2.3 ± 0.2		1051 ± 105	12 h	-		-
24 h	0.37 ± 0.04		169 ± 17	24 h	-		-
48 h	0.15 ± 0.02		70 ± 7	48 h	0.10 ± 0.01		45 ± 4

**Table 3:** Experimental conditions used to synthesize AuNPs and AuNP–MTX conjugates.

				Ratio Au/MTX/TCS			
	HAuCl_4_	MTX	TCS	HAuCl_4_	MTX	TCS	pH
		mmol					
AuNPs	0.025	-	0.55	1	-	0.045	8.77
AuNP–MTX200	0.025	0.0020	0.55	1	0.2	0.045	7.47
AuNP–MTX300	0.025	0.0030	0.55	1	0.3	0.045	7.65
AuNP–MTX400	0.025	0.0040	0.55	1	0.4	0.045	7.72
